# *Pseudogymnoascus destructans:* Causative Agent of White-Nose Syndrome in Bats Is Inhibited by Safe Volatile Organic Compounds

**DOI:** 10.3390/jof4020048

**Published:** 2018-04-10

**Authors:** Sally Padhi, Itamar Dias, Victoria L. Korn, Joan W. Bennett

**Affiliations:** Department of Plant Biology, Rutgers University, 59 Dudley Road, New Brunswick, NJ 08901, USA; itamarbraga@gmail.com (I.D.); victoria.korn@rutgers.edu (V.L.K.); profmycogirl@yahoo.com (J.W.B.)

**Keywords:** bats, white-nose syndrome (WNS), *Pseudogymnoascus destructans*, volatile organic compounds (VOCs), 1-octen-3-ol (mushroom alcohol), (*R*)-(−)-1-octen-3-ol (*R* form), (*S*)-(+)-1-octen-3-ol (*S* form), *trans*-2-hexenal (leaf aldehyde)

## Abstract

White-nose syndrome (WNS) is caused by *Pseudogymnoascus destructans*, a psychrophilic fungus that infects hibernating bats and has caused a serious decline in some species. Natural aroma compounds have been used to control growth of fungal food storage pathogens, so we hypothesized that a similar strategy could work for control of *P. destructans*. The effectiveness of exposure to low concentrations of the vapor phase of four of these compounds was tested on mycelial plugs and conidiospores at temperatures of 5, 10 and 15 °C. Here we report the efficacy of vapor phase mushroom alcohol (1-octen-3-ol) for inhibiting mycelial and conidiospore growth of *P. destructans* at 0.4 and 0.8 µmol/mL and demonstrate that the R enantiomer of this compound is more effective than the S enantiomer, supporting the finding that biological systems can be sensitive to stereochemistry. Further, we report that vapor phase leaf aldehyde (*trans*-2-hexenal), a common aroma compound associated with cut grass odors and also the major volatile compound in extra virgin olive oil, is more effective than mushroom alcohol. At 0.05 µmol/mL, *trans*-2-hexenal is fungicidal to both conidiospores and mycelia of *P. destructans*.

## 1. Introduction

White-nose syndrome (WNS), a fungal disease of hibernating bats, has decimated bat populations in North America [1,2] and threatens the extinction of several bat species [3]. WNS probably was introduced to North America from Europe where the disease is endemic but where bats appear to be resistant [4]. The steep declines in North American bat populations make WNS perhaps the most devastating mammalian wildlife disease in recent history [5]. For some bat species, population sizes have declined 99% in WNS-infected hibernacula [3,6,7].

WNS is caused by *Pseudogymnoascus destructans* (formerly known as *Geomyces destructans),* a cold-loving fungus with growth restricted to temperatures of approximately 3–15 °C and >90% relative humidity [8,9]. During hibernation, the body temperature of bats ranges from 2–15 °C which is similar to the optimum growth temperature for the fungus [10]. The disease infects the cutaneous tissues of bats, producing a white-colored fungal growth on the muzzle and wings [11]. Hibernating bats arouse more frequently from torpor [12,13,14] resulting in depletion of fat reserves, emaciation and death [15]. WNS pathology also includes changes in electrolyte balance and hydration [11,16], chronic respiratory acidosis [17], immune response [18] and oxidative stress [19].

There is a strong need to discover control measures to control *P. destructans* and several studies have shown promise. For example, in *in vitro* research, the growth of *P. destructans* was inhibited by volatile compounds made by the bacterium *Rhodococcus rhodochrous* [20,21] and *trans*, *trans*-farnesol, a sesquiterpene made by the yeast *Candida* [22]. Moreover, some preliminary data from our laboratory showed that volatile phase racemic 1-octen-3-ol (mushroom alcohol) could retard the mycelial growth of *P. destructans*, with exposure to 0.8 µmol/mL being fungicidal and exposure to 0.08 μmol/mL being fungistatic [23]. This common fungal volatile exists as two optical isomers or enantiomers: (*R*)-(−)-1-octen-3-ol and (*S*)-(+)-1-octen-3-ol. Chiral discrimination is important in the activity of many biosystems [24], so we have tested the effect of volatilized R- and S- enantiomers of 1-octen-3-ol on the growth of *P. destructans* mycelial plugs and conidiospores.

Plant pathologists have studied a number of generally recognized as safe (GRAS) volatile compounds for use as postharvest fumigants to control mold pathogens in stored fruits and vegetables [25,26,27]. There are many physiological similarities between plant and animal pathogenic fungi [28,29] so we hypothesized that compounds that worked against common plant pathogenic fungi might also be effective against *P. destructans*. We focused on *trans*-2-hexenal, an important aroma compound of green plants also known as leaf aldehyde, which is known to have pronounced antimicrobial effects [30,31,32,33].

Our fumigation study has been conducted on both mycelia and conidiospores of *P. destructans*. Our long-term aim is to find an antifungal fumigant that will prevent or retard the growth of propagules of this serious bat pathogen but not harm either the hibernating bat or the cave ecosystem. Our immediate goals are as follows: (1) to compare the effects of four concentrations of *trans*-2-hexenal with the R, S and racemic forms of 1-octen-3-ol on the growth of *P. destructans* at 5, 10 and 15 °C; (2) to determine if the growth-inhibiting properties of the four volatilized substances would continue after the fungus is removed from the presence of the VOCs; and (3) having shown that *trans*-2-hexenal is far more effective than 1-octen-3-ol in inhibiting growth of both mycelia and conidiospores of *P. destructans*, to test the response of *P. destructans* to extremely low concentrations (0.01, 0.02 and 0.05 µmol/mL) of this six carbon aldehyde for use in implementation of a possible fumigation strategy in bat hibernacula.

## 2. Materials and Methods

### 2.1. Chemicals

**Figure 1:**
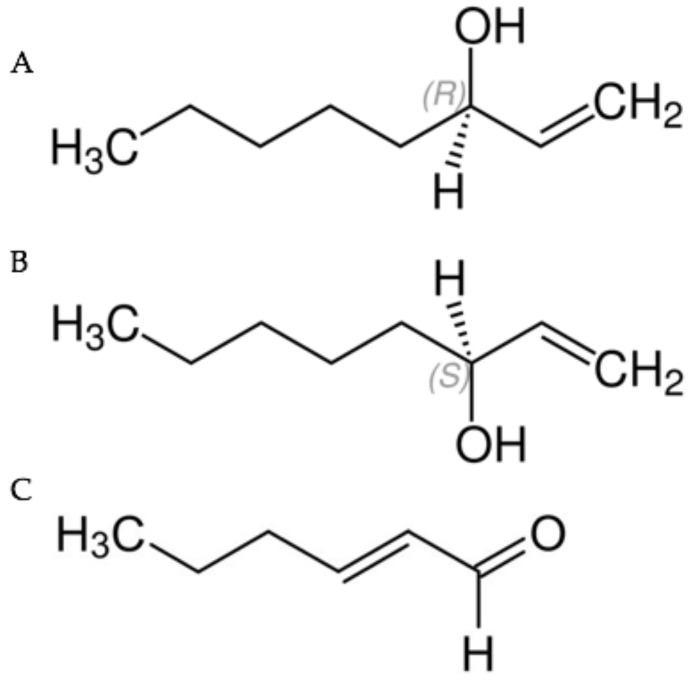
(**A**) (*R*)-(−)-1-octen-3-ol (*R* form); (**B**) (*S*)-(+)-1-octen-3-ol (*S* form); (**C**) *trans*-2-hexenal.

Chemical standards of liquid phase racemic 1-octen 3-ol (synonym mushroom alcohol) and *trans*-2-hexenal (synonyms include *trans*-2-hexen-1-al, (*E*)-2-Hexenal, (*E*)-Hex-2-enal and leaf aldehyde) were purchased from Sigma-Aldrich (St. Louis, MO, USA). The enantiomers (*R*)-(−)-1-octen-3-ol (*R* form) and (*S*)-(+)-1-octen-3-ol (*S* form) were gifts from Bedoukian Research, Danbury, CT, USA. The chemical structures of leaf aldehyde and the *R*- and *S*- forms of mushroom alcohol are illustrated in Figure 1.

### 2.2. Fungal Strains and Media

*Pseudogymnoascus destructans*, (MYA-4855TM) was obtained from the American Type Culture Collection, Manassas, VA, USA. Throughout our work, *P. destructans* was handled according to all procedures required for Level 2 classification pathogens. All exposure experiments were conducted with *P. destructans* grown on Potato Dextrose Agar (PDA) (Difco, Becton, Dickinson & Company, Sparks, MD, USA). For the preparation of conidiospores and viable counts of conidiospore concentrations, Sabouraud Dextrose Agar (SDA) (Difco), supplemented with 200 mg/L MnSO_4,_ was used.

### 2.3. Preparation of Fungal Inocula

To obtain mycelial plugs, fungi were cultured at 15 °C on PDA for three weeks. Using a #3 cork borer, mycelial plugs were taken from the actively growing outer edge of 21 day old colonies. To obtain conidiospores of *P. destructans*, cultures were incubated at 15 °C for 21 days on SDA. The conidiospores were harvested by adding 10 mL of CHS (Conidia Harvesting Solution) to each plate and gently scraping the fungal growth with an inoculation loop to help release the spores. CHS was composed of 0.05% Tween 80% and 0.9% NaCl. The suspension was then filtered through glass wool and the flow through was centrifuged at 5000 rpm for 15 min. The supernatant was removed and the conidiospore pellet was re-suspended in 10 mL of PBS (phosphate-buffered saline) solution at pH 7.0 [20,21].

### 2.4. Volatile Exposures 

Plastic split Petri plates (100 × 15 mm), also known as I-plates, were used. One half of the plate contained 10 mL of PDA and the other half contained a sterile glass cover slip (22 × 22 mm) for the placement of liquid aliquots of the VOC being tested. The amounts of liquid VOC needed were calculated according to the molarity of the VOC and the volume of the Petri plate. For example, in the highest concentration of *trans*-2-hexenal tested (1.0 µmol/mL), 0.0071 mL of the liquid standard was placed on a cover slip and allowed to volatilize in the 60 mL of air space available in the Petri dish.

Mycelial plugs of 21 day cultures were subcultured onto fresh PDA plates and exposed to vapors of 0.05, 0.1, 0.5 or 1.0 µmol/mL of *trans*-2-hexenal, or 0.04, 0.08, 0.4 or 0.8 µmol/mL of racemic 1-octen-3-ol, *R*-1-octen 3-ol or *S*-1-octen 3-ol. Inoculated plates were sealed with two layers of Parafilm. Both 1-octen-3-ol and *trans*-2-hexenal have a distinctive odor so in order to determine if there was a loss of VOCs from the Petri plates, each VOC treatment was placed in two-liter glass containers with tightly fitting propylene lids. The sealed plates were removed weekly from the two-liter glass containers in order to record growth measurements. The lack of odor indicated a minimal loss of VOCs from the sealed Petri plates. The sealed plates were not opened when the measurements were made so as not to compromise the headspace within the Petri plates.

Plates were incubated at 5, 10 or 15 °C for three weeks and growth measurements were made weekly. The amount of mycelial growth was recorded by averaging two diameter measurements taken on each colony at right angles of each mycelial plug. In a set of parallel VOC exposure experiments conducted at 10 °C, suspensions of conidiospores were spread on I-plates and grown in a shared atmosphere with the four different VOCs using the same parameters as with mycelial plugs.

A second exposure system was designed which would allow the testing of *trans*-2-hexenal on spore growth in concentrations smaller than 0.05 µmol/mL by increasing the volume of the exposure system. Three 60 mm Petri plates per treatment were inoculated with conidiospores, the covers removed and the plates placed in two-liter glass containers with tightly fitting propylene lids. *trans*-2-hexenal was added to the glass containers so that when vaporized the conidiospores would be exposed to 0.005, 0.01, 0.02 or 0.05 µmol/mL of *trans*-2-hexenal. The plates were incubated for 3 weeks at 10 °C.

### 2.5. Subculture of VOC Treated P. destructans

After three weeks, in order to determine whether the action of inhibitory VOCs was fungicidal or fungistatic, the mycelial plugs or media containing conidiospore suspensions were removed from the presence of volatilized test compounds, transferred to fresh PDA media, sealed with a double layer of Parafilm and placed inside clean, VOC-absent glass containers. The transferred mycelial plugs were incubated for three additional weeks at 5, 10 or 15 °C. Three replicates were used per treatment and the experiments were repeated twice. At the end of the three-week period, plates containing mycelial plugs were photographed using a Sony DSC-H9 81 mega pixel camera (Sony Corporation, New York, NY, USA) and a Bio-Rad Universal Hood II with a CCD (couple-charged device) camera using Chemi Doc XRS software (Bio-Rad Laboratory Inc., Hercules, CA, USA). Quantitative data were analyzed using Excel software (Microsoft, Redmond, WA, USA) and Sigma Plot (SPSS Science Inc., Chicago, IL, USA). Error bars indicate standard error (SE) of the mean.

In the second exposure system designed to test *trans*-2-hexenal in amounts less than 0.05 µmol/mL, plates containing VOC treated spores were removed from VOC exposure and covered with sterile fresh covers. The plates were incubated for three more weeks at 10 °C. Three replicates were used per treatment and the experiments were repeated twice. When no growth was observed after three weeks, plates were incubated for another 12 weeks in the absence of VOCs in order to verify that the fungus was no longer viable (i.e., that the VOC exposure was fungicidal).

## 3. Results

### 3.1. Growth of VOC Treated Mycelial Plugs

**Figure 2:**
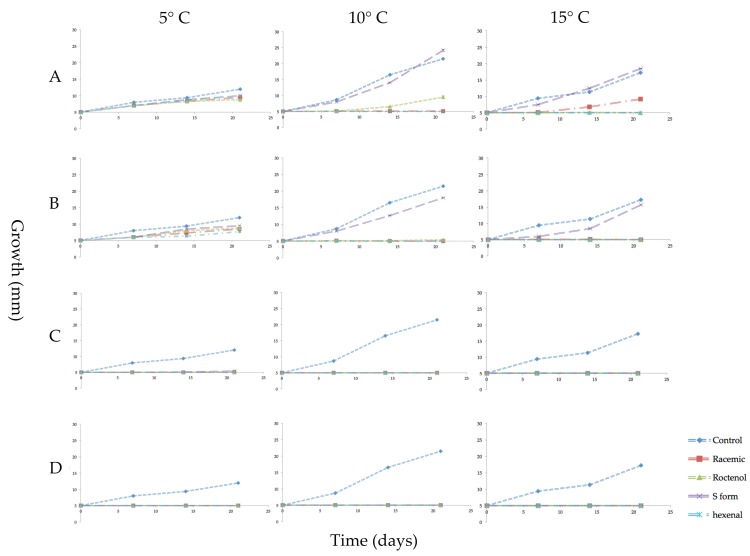
Growth in millimeters (mm) of mycelial plugs of *Pseudogymnoascus destructans* exposed for 3 weeks to vapors of racemic 1-octen-3-ol, (*R*)-(−)-1-octen-3-ol (*R* form), (*S*)-(+)-1-octen-3-ol (*S* form), or *trans*-2-hexenal and cultured at 5, 10 or 15 °C. Error bars indicate the standard error of the mean. VOC treatments as follows: (**A**) 0.04 µmol/mL of the three forms of 1-octen-3-ol or 0.05 µmol/mL of *trans*-2-hexenal; (**B**) 0.08 µmol/mL of 1-octen-3-ol or 0.1 µmol/mL *trans*-2-hexenal; (**C**) 0.4 µmol/mL 1-octen-3-ol or 0.5 µmol/mL *trans*-2-hexenal; (**D**) 0.8 µmol/mL 1-octen-3-ol or 1.0 µmol/mL *trans*-2-hexenal.

**Figure 3:**
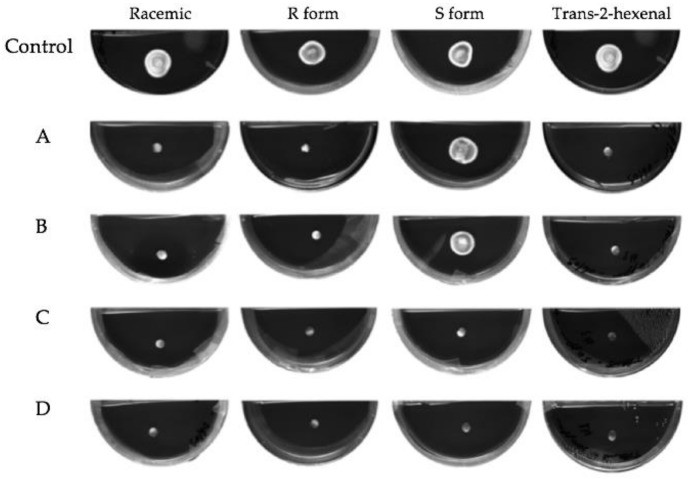
Mycelial plugs of *Pseudogymnoascus destructans* exposed to vapors of for 3 weeks at 15 °C. VOC treatments as follows: (**A**) 0.04 µmol of the three forms of 1-octen-3-ol or 0.05 µmol of *trans*-2-hexenal; (**B**) 0.08 µmol/mL of 1-octen-3-ol or 0.1 µmol *trans*-2-hexenal; (**C**) 0.4 µmol/mL 1-octen-3-ol or 0.5 µmol/mL *trans*-2-hexenal; (**D**) 0.8 µmol/mL 1-octen-3-ol or 1.0 µmol/mL *trans*-2-hexenal.

The effect on the growth of mycelial plugs of *P. destructans* at 5, 10 or 15 °C from exposure to four concentrations of volatile phase *trans*-2-hexenal or the three forms of 1-octen-3-ol is shown in graphed form in Figure 2. Controls grew best at 10 °C with an average increase in mycelial diameter of 200 mm after three weeks. At 15 °C, controls increased about 130 mm, while at 5 °C they increased in diameter about 50 mm during the three-week period. At all three temperature regimes, exposure to the higher concentrations of the four VOCs inhibited growth. The *S* form of 1-octen-3-ol caused very little growth inhibition at 0.04 and 0.08 µmol/mL. At 10 and 15 °C, 0.05 and 0.1 µmol/mL *trans*-2-hexenal were more effective than 1-octen-3-ol in inhibiting mycelial growth of *P. destructans* (Figure 2). For all treatments, incubation of the fungus at 5 °C reduced mycelial growth and also reduced the effectiveness of the VOCs. As shown in Figure 2, at 5 °C, exposure to the two lowest concentrations of the VOCs did not completely inhibit growth; only the two highest concentrations tested inhibited *P. destructans* at this temperature. Figure 3 shows images of the mycelial plugs after three weeks of exposure to these treatments at 15 °C. The mycelial growth of controls and the mycelial growth of plugs exposed to the 0.04 and 0.08 µmol/mL of the *S* form of 1-octen-3-ol are similar, while the R enantiomer and racemic form of 1-octen-3-ol showed growth inhibition, as did the mycelial plugs exposed to *trans*-2-hexenal at 0.05, 0.1, 0.5 and 1.0 µmol/mL.

### 3.2. Subculture of Mycelial Plugs after VOC Treatment

**Figure 4:**
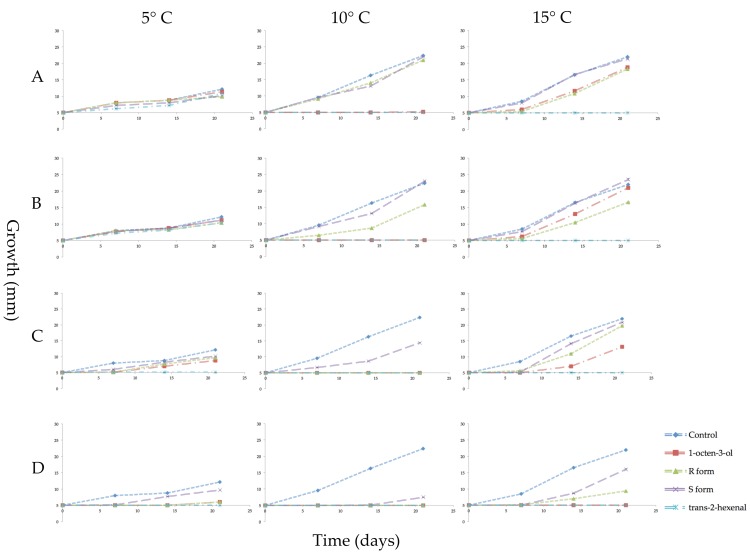
Growth in millimeters (mm) of mycelial plugs of *Pseudogymnoascus destructans* which were subcultured in ambient air for 3 weeks at 5, 10, or 15 °C after prior exposure for 3 weeks to vapors of racemic 1-octen-3-ol, (*R*)-(−)-1-octen-3-ol (*R* form), (*S*)-(+)-1-octen-3-ol (*S* form), or *trans*-2-hexenal. Error bars indicate the standard error of the mean. VOC treatments as follows: (**A**) 0.04 µmol/mL of the three forms of 1-octen-3-ol or 0.05 µmol/mL of *trans*-2-hexenal; (**B**) 0.08 µmol/mL of 1-octen-3-ol or 0.1 µmol/mL *trans*-2-hexenal; (**C**) 0.4 µmol/mL 1-octen-3-ol or 0.5 µmol/mL *trans*-2-hexenal; (**D**) 0.8 µmol/mL 1-octen-3-ol or 1.0 µmol/mL *trans*-2-hexenal.

After the three-week VOC exposure, all mycelial plugs were subcultured to new PDA media and incubated for three weeks in the absence of VOCs to determine if the treatments had killed the mycelia (fungicidal effect) or merely inhibited their growth (fungistatic effect). All treatments of *trans*-2-hexenal at 10 and 15 °C were fungicidal. At the lower temperature of 5 °C, cultures exposed to 0.05 and 0.1 µmol/mL of *trans*-2-hexenal showed some growth indicating a fungistatic effect at these two lower concentrations. With the 1-octen-3-ol treatments, the *S* form was least effective with recovery of mycelial growth at all four concentrations and three temperatures tested (Figure 4). At 10 °C, 0.08, 0.4 and 0.8 µmol/mL racemic 1-octen-3-ol was fungicidal while 0.04 µmol/mL was fungistatic. Microscopic examination at the end of the incubation period showed some hyphal extensions although this growth was not measurable macroscopically. At 10 °C, the *R* form of 1-octen-3-ol was fungicidal at 0.4 and 0.8 µmol/mL. At 15 °C, 0.8 µmol/mL of racemic 1-octen-3-ol was fungicidal while the *R* form was fungistatic. At 5 °C, mycelial plugs exposed to 0.8 µmol/mL of either the racemic or the *R* form showed some recovery after three weeks incubation.

### 3.3. Effect of VOCs on Growth from Conidiospores at 10 °C

**Figure 5:**
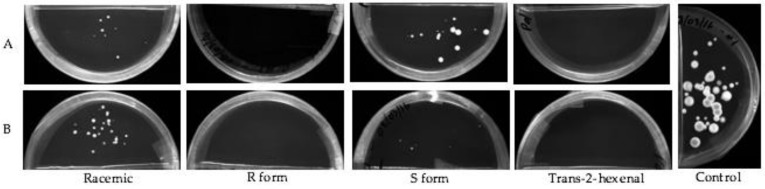
The effect of exposure to low concentrations of volatilized racemic 1-octen-3-ol, (*R*)-(−)-1-octen-3-ol (*R* form), (*S*)-(+)-1-octen-3-ol (*S* form) and *trans*-2-hexenal on growth of *P. destructans* conidiospores after 3 weeks of incubation at 10 °C. VOC treatments as follows: (**A**) 0.04 µmol/mL of three enantiomers of 1-octen-3-ol or 0.05 µmol/mL of *trans*-2-hexenal; (**B**) 0.08 µmol/mL of three enantiomers of 1-octen-3-ol or 0.1 µmol/mL of *trans*-2-hexenal.

The effect of exposure to the volatile phase of these VOCs on growth from conidiospores was studied at 10 °C. After incubation for three weeks at 10 °C, conidiospores exposed to 0.04 µmol/mL and 0.08 µmol/mL of racemic 1-octen-3-ol, or its S isomer, gave rise to a few scattered colonies, however no growth was observed for conidiospores exposed to the R enantiomer (Figure 5). At higher concentrations (0.4 and 0.8 µmol/mL) all three forms of 1-octen-3-ol inhibited conidiospore germination. *trans*-2-hexenal was more effective than 1-octen-3-ol, since no growth from conidiospores was observed at any of the volatile exposures tested (0.05, 0.1, 0.5 or 1.0 µmol/mL). When the plates were removed from the presence of volatiles and incubated for 21 days, conidiospores exposed to 0.08 µmol/mL of the R enanatiomer of 1-octen-3-ol produced colonies. In contrast, no growth was observed for conidiospores exposed to *trans*-2-hexenal at any of the concentrations tested.

### 3.4. Effect of 0.005, 0.01, 0.02 and 0.05 µmol/mL trans-2-hexenal on Conidiospore Growth at 10 °C

**Figure 6:**
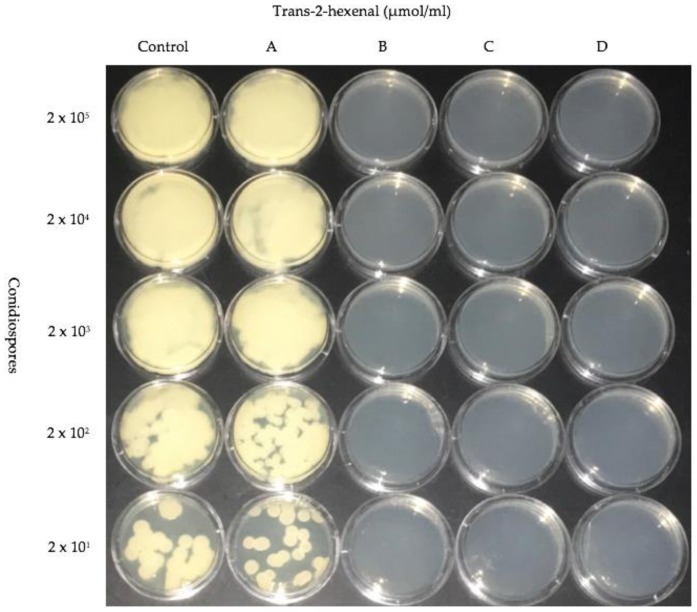
Dilution assay of five different concentrations of *P. destructans* conidiospores treated with *trans*-2-hexenal. Vapors of VOC as follows: (**A**) 0.005 µmol/mL; (**B**) 0.01 µmol/mL; (**C**) 0.02 µmol/mL; (**D**) 0.05 µmol/mL.

In order to determine if exposure to lower concentrations of *trans*-2-hexenal were also inhibitory to the germination of *P. destructans* conidiospores, a new exposure system was designed that would allow the testing of lower concentrations of this compound. *trans*-2-hexenal is not water soluble and we have found that non-polar solvents that can be used to dissolve it also have independent effects on the growth of *P. destructans*. Therefore, in order to accurately test lower concentrations of *trans*-2-hexenal on conidiospore germination, we increased the volume of our exposure system. In these experiments, a serial dilution of *P. destructans* conidiospores was exposed to *trans*-2-hexenal vapors in concentrations of 0.005, 0.01, 0.02 and 0.05 µmol/mL. The three higher concentrations of exposure prevented growth from conidispores, however conidiospores exposed to 0.005 µmol/mL of *trans*-2-hexenal resumed growth (Figure 6). After a three-week period of incubation, the treated plates were removed from *trans*-2-hexenal exposure and incubated for another 21 days in the absence of *trans*-2-hexenal. Conidiospores that had been previously exposed to 0.01 and 0.02 µmol/mL of *trans*-2-hexenal resumed growth. Conidiospores formerly treated with 0.05 µmol/mL *trans*-2-hexenal did not resume growth, even after two months, so this concentration was fungicidal *to P. destructans.*

## 4. Discussion

In addition to the serious negative effects of *P. destructans* on hibernating bats, other fungal pathogens have caused several recent epizootics among vertebrates including *Batrachochytrium dendrobatitis,* cause of chytridiomycosis in frogs [34], and *Ophidiomyces ophiodiicola,* cause of snake fungal disease in snakes [35]. There is a pressing need to find environmentally benign antifungal compounds to treat the fungi that cause these devastating wild life diseases.

Volatile organic compounds (VOCs) are small molecules with high vapor pressure that exist in the gaseous state at room temperature [36]. Many of the volatile compounds from natural sources, such as those found in essential oils, have documented antimicrobial activities and are generally recognized as safe (GRAS) by the US Food and Drug Administration [26,33]. For example, at low concentrations, vapors of both hexanal and octanal completely inhibited the radial growth of *Aspergillus parasiticus* [37]. Similarly, vapors of racemic 1-octen-3-ol and *trans*-2-hexenal were effective against the mycelial growth of *Penicillium chrysogenum* [38]. Moreover, exposure to *trans*-2-hexenal, *trans*-2-hexen-1-ol, cis-3-hexen-1-ol and 1-hexanol inhibited growth of *Fusarium avenaceum* and *Fusarium graminearum* [30]; and *trans*-2-hexenal inhibited growth of *Rhizoctonia solani* and *Sclerotium rolfsii* [39]. We postulated that *trans*-2-hexenal would be effective against *P. destructans*.

In a previous preliminary study, we tested the eight-carbon alcohol 1-octen-3-ol, and found that when exposed at 15 °C, low concentrations (0.4 and 0.8 μmol/mL) of racemic 1-octen-3-ol inhibited growth of *P. destructans* [23]. Mushroom alcohol is a chiral compound and biological systems are known to be sensitive to stereochemistry [24,40]. Therefore, in this current report, we tested to see if the inhibitory effects of the racemic, R-and S- forms of this chiral compound would be different. Indeed, we showed that both racemic 1-octen-3-ol and the *R* form were more effective than the *S* form. Moreover, the relative impacts of the three forms of vapors of mushroom alcohol were temperature dependent. When cultures grown at 5 °C were exposed to low concentrations of 1-octen-3-ol vapors, the effectiveness of all three forms was reduced, likely due to reduced volatility at this low temperature. Because we did not quantitatively monitor the amount of VOC present during the duration of experiments, we recognize that the amount of VOC we report in our exposure figures are the initial concentrations of exposure, that is, maximum amounts. Some loss of VOC likely occurred during the course of the experiments, although we did not detect the distinctive odors of mushroom alcohol or leaf aldehyde.

At low concentrations, the vapors of 1-octen-3-ol were not fungicidal. Rather, mushroom alcohol functioned to retard growth from mycelia and conidiospores. Our data support research on several other fungal species demonstrating the spore germination inhibiting properties of mushroom alcohol, including *Agaricus bisporus, Aspergillus nidulans* and *Penicillium paneum* [41,42,43].

*trans*-2-hexenal was more effective than 1-octen-3-ol for inhibiting growth from mycelial plugs and conidiospores of *P. destructans.* It completely inhibited growth of mycelial plugs and growth from conidiospores at 0.05 µmol/mL. Unlike 1-octen-3-ol, where mycelial growth resumed when the colonies were removed from the presence of the VOC and growth from conidiospores was observed, *P. destructans* exposed to 0.05 µmol/mL of *trans*-2-hexenal showed growth from neither mycelia nor conidiospores indicating that vapors of *trans*-2-hexenal were fungicidal at this concentration. Commonly known as leaf aldehyde, *trans*-2-hexenal is widely distributed in plants [44] where it is a major constituent of the odor of newly mown grass [45]. *trans*-2-hexenal is also the major VOC in extra virgin olive oil [46,47], where it has been reported to have broad antimicrobial effects [48] and contributes to the ability of olive oil to inhibit the growth of medically important fungi such as *Trichophyton mentagrophytes, Candida* and *Microsporum canis* [49]. *trans*-2-hexenal is an approved food additive by the US Food and Drug Administration [26]. Interestingly, there is also evidence that this compound may have a positive impact on rodent physiology. In controlled laboratory experiments, a mixture of *trans*-2-hexenal and cis-3-hexanol relieved stress markers in rats [50,51].

Temperature was an important parameter for both the growth of the fungus and the effectiveness of the volatile treatments. In our experiments, control cultures of *P. destructans* grew best at 10 °C. Blehart et al. [10] reported an optimal growth temperature of *P*. *destructans* between 5 °C and 10 °C, with only marginal growth above 15 °C. We found that the efficacy of the volatile treatments in inhibiting growth was less pronounced at 5 °C than at the higher temperatures of 10 °C and 15 °C. The effectiveness of VOCs has been reported to be dependent on their vapor pressure, with higher temperatures enhancing their effectiveness [32,52,53]. If adopted for use in hibernacula with cold temperatures, it would be important to use appropriate concentrations that take into account the lower efficacy at 5 °C.

Because WNS is associated with hibernation in caves and other enclosed spaces, we envision an intervention whereby bat hibernacula are fumigated with low concentrations of leaf aldehyde (*trans*-2-hexenal) in such a fashion as to reduce the load of *P. destructans*. We are currently developing methods for formulating *trans*-2-hexenal for use in scaled up model habitats and devising methods for testing its toxicity in mammalian tissues.
